# Crenobiont, stygophile and stygobiont molluscs in the hydrographic area of the Trebišnjica River Basin

**DOI:** 10.3897/zookeys.1047.64034

**Published:** 2021-06-28

**Authors:** Andrzej Falniowski, Brian Lewarne, Aleksandra Rysiewska, Artur Osikowski, Sebastian Hofman

**Affiliations:** 1 Department of Malacology, Institute of Zoology and Biomedical Research, Jagiellonian University, ul. Gronostajowa 9, 30-387 Kraków, Poland; 2 The Devon Karst Research Society, Library & Office, 46, Morley Court, Western Approach, Plymouth, Devon, UK; 3 Department of Animal Reproduction, Anatomy and Genomics, University of Agriculture in Krakow, al. Mickiewicza 24/28, 30-059 Kraków, Poland; 4 Department of Comparative Anatomy, Institute of Zoology and Biomedical Research, Jagiellonian University, ul. Gronostajowa 9, 30-387 Kraków, Poland

**Keywords:** Balkans, Bosnia and Herzegovina, cave, COI, H3, karst area, meiofauna, molecular systematics, new genus, new species, spring

## Abstract

In the paper the crenobiont, stygophile and stygobiont malacofauna of the karst area of Popovo Polje around Trebinje (Eastern Herzegovina, BiH) is presented. The materials were collected from springs, caves and interstitial habitats (with a Bou-Rouch pump) at 23 localities. The following species were found: Pisidium cf. personatum A.W. Malm, 1855, *Theodoxus callosus* (Deshayes, 1833), *Sadleriana fluminensis* (Küster, 1852), *Radomaniola curta* (Küster, 1852), Radomaniola cf. bosniaca (Radoman, 1973), *Kerkia briani* Rysiewska & Osikowski, 2020, *Montenegrospeum bogici* (Pešić & Glöer, 2012), *Litthabitella chilodia* (Westerlund, 1886), *Travunijana vruljakensis* Grego & Glöer, 2019, a new genus and species of the Sadlerianinae, *Emmericia ventricosa* Brusina, 1870, Iglica cf. absoloni (A.J. Wagner, 1914), *Plagigeyeria tribunicae* Schütt, 1963, *Paladilhiopsis arion* Rysiewska & Osikowski, 2021, *Valvata montenegrina* Glöer & Pešić, 2008, *Radix labiata* (Rossmässler, 1835), *Galba truncatula* (O. F. Müller, 1774), *Ancylus recurvus* Martens, 1783, *Ancylus* sp. and the amphibiotic Succinea cf. putris (Linnaeus, 1758). The redescription of the genus *Travunijana* Grego & Glöer, 2019, applying the characteristics of shell, female reproductive organs and penis, is also presented. The new genus and species are described, based on the shell, penis, radula and fragmentary data on the female reproductive organs. For all species, the mitochondrial cytochrome oxidase subunit I (COI) is applied to confirm the determination; in the case of *Travunijana* and the new genus, the nuclear histone H3 locus is also used, in order to infer both their distinctiveness and phylogenetic relationships.

## Introduction

The Dinaric Karst is a global hotspot for subterranean biodiversity. This is particularly true in the case of its stygobiont, stygophilic and crenobiont communities. The present paper focusses on providing further evidence of one generally under-reported aspect of freshwater aquatic biodiversity – namely the malacofauna of the Trebišnjica River Basin, predominantly in the hydrographically complex karst area of Eastern Herzegovina in Bosnia and Herzegovina.

The study reported below, was undertaken under the remit of the RS-Bosnia and Herzegovina Official Government Licence, which is granted annually to the “Proteus Project in Bosnia and Herzegovina” to undertake its objective of protecting and conserving endangered cave fauna and by extension, to protect and conservation-manage the natural karst conduit-aquifer hypogean ecosystems containing the endangered cave faunal species. One of the objectives of the Project is to fully characterise these ecosystems and in doing so, to provide an inventory of their biodiversity.

In this context, the contribution made by the visiting team of malacologists from the Department of Malacology of the Jagiellonian University’s Institute of Zoology and Biomedical Research and from Department of Animal Reproduction, Anatomy and Genomics of University of Agriculture in Krakow, both in Poland, has provided the “Proteus Project” with vital information on the biological characteristics and geographic distribution of a range of genera and species of malacofauna collected at 23 locations connected to 11 separate karst conduit-aquifer ecosystems across a wide area of the Trebišnjica River Basin. The 23 sampling locations were purposely selected by the Director of the “Proteus Project” to represent a typical range of karst hydrological features, such as cave resurgence springs (vrelo), ponors and estavelles, either underground or at surface outlets or inlets.

Speleomalacological research on this scale and in such an integrated form, has never been undertaken before now in Bosnia and Herzegovina. Not surprisingly, therefore, the Polish team has identified a new genus and species of meiofaunal gastropod (Mollusca). As a standalone account, these first results, containing verifiable genomic data are of great scientific importance in their own right, but when combined with the associated variety of environmental data being amassed by the “Proteus Project”, they assume a much greater value.

In regard to both ecosystem services and as a nutrient-rich food supply, the importance of the position of malacofauna near the bottom of the “foodchain” of the subterranean aquatic ecosystem, cannot be overstated. Without them being present in all their wonderful variety and population numbers, the diversity of many of the higher cave animals would certainly not be as great.

## Material and methods

**Figure 1.**
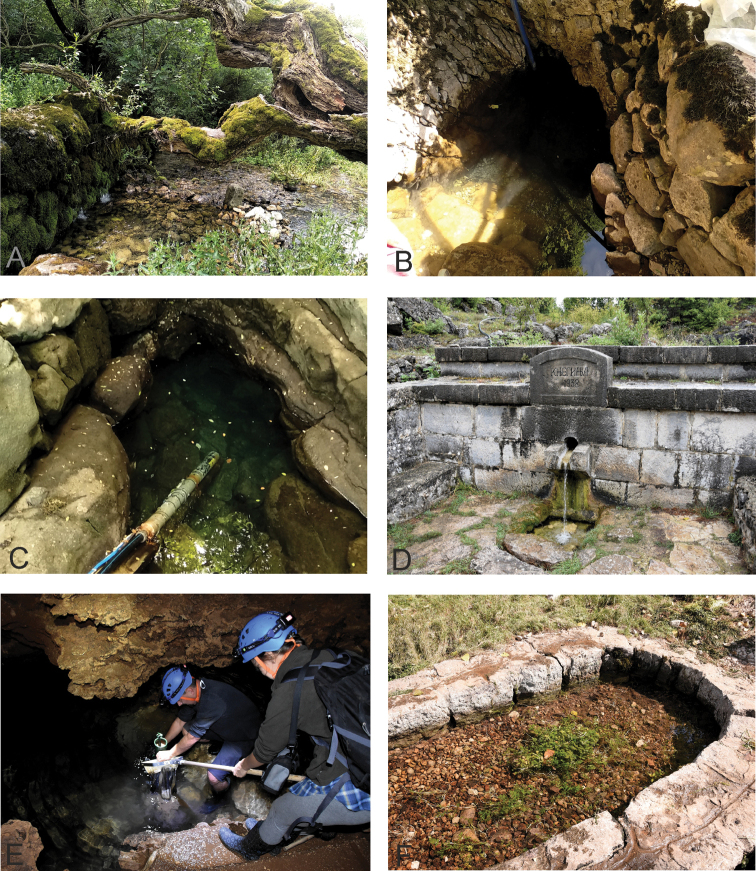
Selected studied localities from Trebinje area, part 1 **A** locality 1, Vrelo „Vrijeka” (Bijeljani), Dabarsko Polje **B** locality 5, Vrelo „Pokrivenik” (Muhareva Ljut), Popovo Polje **C** locality 6, Vrelo „Lukavac” (Zavala) **D** locality 9, Izvor „Knez” (Trklja) **E** pumping of interstitial fauna at locality 11, Vrelo „Tučevac” (Mostaći) **F** locality 13, Vrelo „Polički Studenac” (Crkvina). See also Table 1.

**Figure 2.**
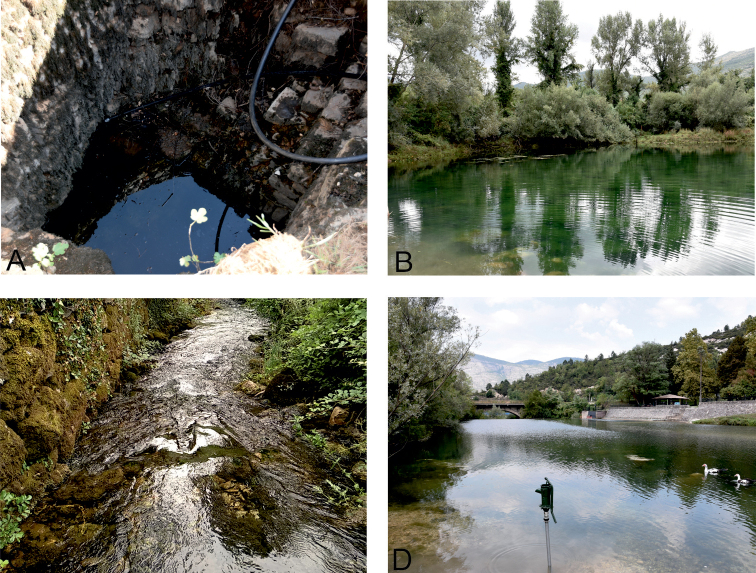
Selected studied localities from Trebinje area, part 2 **A** locality 14, Vrelo “Oko” (Zasad) **B** locality 16, Igorovo Jezero (lake) (Gorica) **C** locality 17, Vrelo „Vruljak 2” (Gorica), Trebinjsko Polje **D** locality 20, confluence of Sušica River and Jazina River (Jazina). See also Table 1.

**Figure 3.**
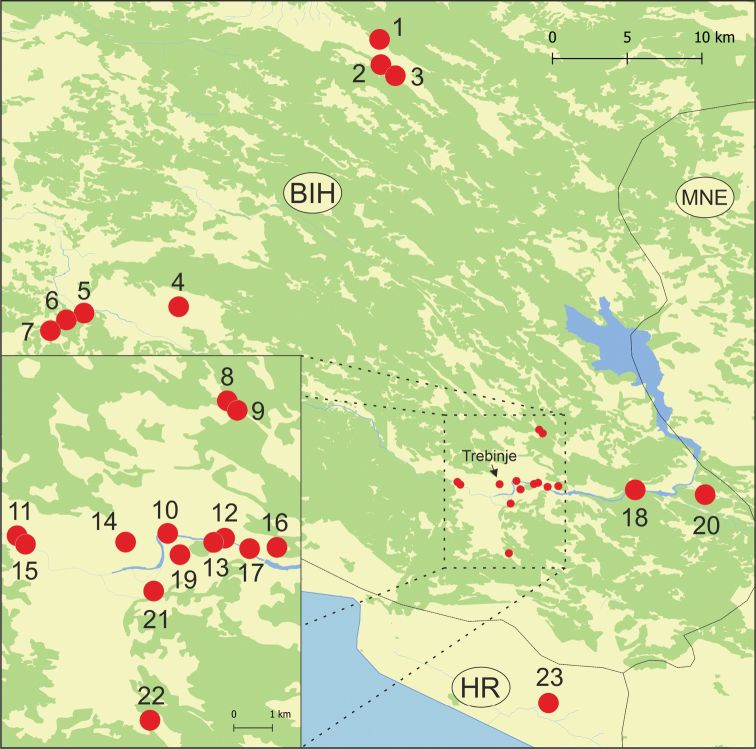
Studied localities.

In June and September 2019, we collected aquatic gastropods from springs, interstitial habitats and caves at 23 localities (Table 1, Figs 1–3). They were either collected by hand and sieve in caves and springs, or with a pump applying the Bou-Rouch technique (Bou and Rouch 1967), to sample interstitial fauna below the sedimented floor of streams, at a depth of about 50 cm. The tube was inserted in the sediment five times, and 20 litres were pumped each time. Samples were sieved through a 500 μm sieve and fixed in 80% analytically pure ethanol, replaced twice, and later sorted. Next, the snails were put in fresh 80% analytically pure ethanol and kept at -20 °C temperature in a refrigerator. Percentages of each identified taxon in each locality are presented in Table 1, with division into samples collected on the surface and with a pump.

**Table 1.** The list of studied localities, with a short description of their characteristics, geographical coordinates and taxa identified.

Id	Site names, characteristics and codes	Coordinates	Taxa confirmed	% of taxa in site (surface/pump)
1	**Vrelo „Vrijeka” (Bijeljani), Dabarsko Polje**; at the outlet (BiH19_08)	43.07417, 18.23899	*Emmericia ventricosa*	0/12.6
	A permanent cave resurgence spring whose water originates from ponors located in Lukavačko Polje.		*Montenegrospeum bogici*	100/0
			Radomaniola cf. bosniaca	0/87.4
2	**Estavela „Ljelješnica”(Bijeljani)**; inside the cave (BiH19_14)	43.05400, 18.24069	–	–
	When checked, this location was hydrologically inactive.			
3	**Rijeka (river) „Vrijeka” (Dabarsko Polje)**; on the surface near entrance of Ponor „Ponikva” (BiH19_15)	43.04535, 18.25217	Radomaniola cf. bosniaca	100/0
	Samples taken under low-flow conditions.			
4	**Estavela „Kapuša” (Dračevo)**; inside the entrance (BiH19_24)	42.85692, 18.07665	–	–
	Checked when the estavelle was hydrologically inactive.			
5	**Vrelo „Pokrivenik” (Muhareva Ljut)**, Popovo Polje; spring at the cave entrance; high water level variation (BiH19_05)	42.85166, 17.99838	*Emmericia ventricosa*	0/100
	Samples taken when the location was hydrologically inactive.			
6	**Vrelo „Lukavac” (Zavala)**; outlet for Vjetrenica Pećina. Spring below the cave entrance; high water level variation (BiH19_06)	42.84643, 17.9846	Radomaniola cf. bosniaca	0/100
	Samples taken when the location was hydrologically inactive.			
7	**Vrelo „Bitomišlje” (Golubinac)**; in valley above Zavala, with Austro-Hungarian infrastructure (BiH19_07)	42.83799, 17.97161	*Litthabitella chilodia*	40.3/0
	Samples taken under extremely low-flow conditions.		*Montenegrospeum bogici*	59.7/0
8	**Izvor „Kneginja” (Trklja)**; a low-flow groundwater spring in Dolomite coming from a limestone blockhouse (BiH19_20)	42.75729, 18.3693	*Ancylus* sp.	0/2.7
			*Litthabitella chilodia*	0/97.3
9	**Izvor „Knez” (Trklja)**; a low-flow groundwater spring in Dolomite coming from a limestone blockhouse (BiH19_21)	42.75463, 18.37218	*Ancylus* sp.	0/2.3
			*Litthabitella chilodia*	0/97.7
10	**Confluence of Trebišnjica River with the Potok (stream) Blace (Blace)**; surface stream from a cave spring-group on the right bank of Trebišnjica River (BiH19_17)	42.71536, 18.35077	*Radomaniola curta*	100/32.1
			*Sadleriana fluminensis*	0/64.3
			Succinea cf. putris	0/2.6
11	**Vrelo „Tučevac” (Mostaći)**; the spring inside the cave (BiH19_13)	42.71445, 18.30278	Radomaniola cf. bosniaca	100/0
	A high-level overflow spring from a locally complex estavelle cave system. When active, its water originates from ponors in Ljubomirsko Polje 14 km away. This was hydrologically inactive when sampled.			
12	**Vrelo „Vruljak 1” (Gorica), Trebinjsko Polje**. This was sampled in the resurgence pool before which 2 cave rivers Rijeka “Goričica” and Rijeka “Vrulje” have joined inside & emerge (BiH19_03)	42.71393, 18.36833	*Emmericia ventricosa*	0/7.8
			Pisidium cf. personatum	50/0
	The cave resurgence spring is just one outlet from a locally very complex cave system, containing a very rich biodiversity. The water originates from ponors in Ljubomirsko Polje about 12 km away.		Radomaniola cf. bosniaca	0/92.2
			*Travunijana vruljakensis*	50/0
13	**Vrelo „Polički Studenac” (Crkvina)**; a cave spring in the left bank of Trebišnjica River (BiH19_11)	42.71288, 18.36514	*Ancylus recurvus*	3.7/0
			*Emmericia ventricosa*	0/44.3
			*Iglicopsis butoti* sp. nov.	27.8/0
			*Kerkia briani*	38.9/0
			*Radomaniola curta*	10.2/7.6
			Radomaniola cf. bosniaca	0/48.1
			*Travunijana vruljakensis*	19.4/0
14	**Vrelo “Oko” (Zasad)**; a spring in the entrance to the cave system; surrounded by ancient limestone-block housing; at the centre of Trebinje (BiH19_23)	42.71274, 18.33697	Radomaniola cf. bosniaca	0/5.9
	This location is permanently hydrologically active and its water originates from ponors in Ljubomirsko Polje 14 km away. Although it is locally regarded as a vrelo, it is actually an estavelle. This was once used as a public water supply.		*Travunijana vruljakensis*	0/94.1
15	**Estavela „Pećine” (Mostaći)** (BiH19_12)	42.71244, 18.30497	*Ancylus recurvus*	100/0
	This is a major estavelle-type outlet for the karst conduit-aquifer originating at the ponors in Ljubomirsko Polje. It was hydrologically inactive when sampled.		*Galba truncatula*	0/100
16	**Igorovo Jezero (lake) (Gorica)**; small lake in a collapsed cave passage with cave springs and containing many ponors; muddy bottom (BiH19_19) The water originates from ponors in both Ljubomirsko Polje and Cibrijansko Polje. The ponors in and around the lake feed water underground downstream to Vrelo “Vruljak 2” (Gorica).	42.71111, 18.38495	*Ancylus* sp.	0/9.1
			*Galba truncatula*	0/36.4
			*Radix labiata*	0/9.1
			*Sadleriana fluminensis*	0/45.4
17	**Vrelo „Vruljak 2” (Gorica)**, Trebinjsko Polje; this location was sampled at the resurgence spring outlet before which 2 cave rivers have joined inside: Rijeka “Pešterčica” and Rijeka “Venator” (BiH19_02) This is a permanently hydrologically active outlet from a locally very complex cave system containing a very rich biodiversity.	42.71062, 18.37618	*Kerkia briani*	15.9/0
			*Plagigeyeria tribunicae*	2.3/0
			*Radomaniola curta*	0/96.5
			*Sadleriana fluminensis*	0/3.5
			*Travunijana vruljakensis*	81.8/0
18	The intermittently active cave spring, **Vrelo „Vražiji Mlin” (D. Grančarevo)**; Trebišnjica Canyon (BiH19_04)	42.70847, 18.44801	Radomaniola cf. bosniaca	0/100
	This is fed by ponors in Jasen Polje. The location is set in dolomitic limestone.			
19	“**Slomljen pecina” (Mokri Dolovi)**; (BiH19_22)	42.70844, 18.35419	–	–
	Since being sampled, this location has now been buried and made inaccessible by urban development.			
20	**Confluence of Sušica River and Jazina River** (Jazina) (BiH19_16)	42.70429, 18.50491	Iglica cf. absoloni	16.7/0
	This was sampled under low-flow conditions. The source of the water is a giant estavelle situated in karstified dolomite with dolomitic limestone.		*Litthabitella chilodia*	83.3/0
			*Radix labiata*	0/72.2
			*Valvata montenegrina*	0/27.8
21	**Vrelo „Lušac” (Gučina)**; at the surface outlet (BiH19_10)	42.70111, 18.3575	*Litthabitella chilodia*	14.6/0
	A permanently hydrologically active outlet from a complex karst conduit-aquifer, whose principal source is unproven. This was once a public water supply.		*Montenegrospeum bogici*	22.0/0
			Pisidium cf. personatum	4.9/0
			*Paladilhiopsis arion*	58.5/0
			*Travunijana vruljakensis*	0/100
22	**Estavela „Mali Šumet” (Bugovina)**, Mokro Polje: in the entrance shaft (BiH19_01)	42.65665, 18.34458	*Emmericia ventricosa*	0/100
	The entrance comprises a neo-circular stone wall leading down into the interior by more than 20 stone steps set into the natural stone floor of the karst conduit. The construction is of Austro-Hungarian origin and designed to give easy access to the potable water supply for local people. The location was hydrologically inactive when sampled.			
23	**River Konavoska Ljuta (Ljuta)**, Croatia; samples from the surface (Stones, plants) (BiH19_18)	42.53408, 18.37647	Pisidium cf. personatum	15.6/0
	This karst river originates from Vrelo “Konavoska Ljuta” a few metres upstream from the sampling location. However, the water itself originates from a ponor 10 km away in Zubačko Polje near Trebinje in Eastern Herzegovina. This cave resurgence spring is used as a public water supply. The samples were collected under low-flow conditions.		*Radomaniola curta*	84.4/100

The shells were photographed with a Canon EOS 50D digital camera, under a Nikon SMZ18 microscope. The dissections were done under a Nikon SMZ18 microscope with dark field, equipped with Nikon DS-5 digital camera, whose captured images were used to draw anatomical structures with a graphic tablet. Morphometric parameters of the shell were measured by one person using a Nikon DS-5 digital camera and ImageJ image analysis software (Rueden et al. 2017). The radulae were extracted with Clorox, applying the techniques described by Falniowski (1990), and examined and photographed using a HITACHI S-4700 scanning electron microscope.

DNA was extracted from whole specimens; tissues were hydrated in TE buffer (3 × 10 min); then total genomic DNA was extracted with the SHERLOCK extraction kit (A&A Biotechnology), and the final product was dissolved in 20 μl of tris-EDTA (TE) buffer. The extracted DNA was stored at -80 °C at the Department of Malacology, Institute of Zoology and Biomedical Research, Jagiellonian University in Kraków (Poland).

Mitochondrial cytochrome oxidase subunit I (COI) and nuclear histone 3 (H3) loci were sequenced. Details of PCR conditions, primers used and sequencing technique were as given in Szarowska et al. (2016a). Sequences were initially aligned in the MUSCLE (Edgar 2004) programme in MEGA 7 (Kumar et al. 2016) and then checked in BIOEDIT 7.1.3.0 (Hall 1999). Uncorrected p-distances were calculated in MEGA 7. Estimation of the proportion of invariant sites and the saturation test (Xia 2000; Xia et al. 2003) were performed using DAMBE (Xia 2018). In the phylogenetic analysis, additional sequences from GenBank were used (Table 2). The phylogenetic analysis was performed applying two approaches: Bayesian Inference (BI) and Maximum Likelihood (ML). The Bayesian analyses were run using MrBayes v. 3.2.3 (Ronquist et al. 2012) with defaults for most priors. Two simultaneous analyses were performed, each with 10,000,000 generations, with one cold chain and three heated chains, starting from random trees and sampling the trees every 1000 generations. The first 25% of the trees were discarded as burn-in. The analyses were summarised as a 50% majority-rule tree. The Maximum Likelihood analysis was conducted in RAxML v. 8.2.12 (Stamatakis 2014) using the RAxML-HPC v.8 on XSEDE (8.2.12) tool via the CIPRES Science Gateway (Miller et al. 2010). We applied the GTR model whose parameters were estimated by RAxML (Stamatakis 2014).

**Table 2.** Taxa used for phylogenetic analyses with their GenBank accession numbers and references.

Species	COI/H3 GB numbers	References
*Agrafia wiktori* Szarowska & Falniowski, 2011	JF906762/MG543158	Szarowska and Falniowski 2011/Grego et al. 2017
*Alzoniella finalina* Giusti & Bodon, 1984	AF367650/-	Wilke et al. 2001
*Anagastina zetavalis* (Radoman, 1973)	EF070616/-	Szarowska 2006
*Ancylus* sp. B	DQ301830DQ301838/-	Albrecht et al. 2006
*Ancylus* sp. C4	KY012232KY012163/-	Macher et al. 2016
*Ancylus* sp. – clade 3	AY350516AY350519/-	Pfenninger et al. 2003
*Ancylus* sp. – clade 4	AY350520AY350521/-	Pfenninger et al. 2003
*Avenionia brevis berenguieri* (Bourguignat, 1882)	AF367638/-	Wilke et al. 2001
*Belgrandia thermalis* (Linnaeus, 1767)	AF367648/-	Wilke et al. 2001
*Belgrandiella kuesteri* (Boeters, 1970)	MG551325/-	Osikowski et al. 2018
*Belgrandiella kusceri* (A. J. Wagner, 1914)	-/MG551366	Osikowski et al. 2018
*Bithynia tentaculata* (Linnaeus, 1758)	AF367643/-	Wilke et al. 2001
*Bracenica gloeri* Grego, Fehér & Erőss, 2020	MT396209/-	Hofman et al. 2020a
*Bythinella cretensis* Schütt, 1980	KT353689/-	Szarowska et al. 2016b
*Bythiospeum acicula* (Hartmann, 1821)	KU341350/MK609536	Richling et al. 2016/Falniowski et al. 2019
*Daphniola louisi* Falniowski & Szarowska, 2000	KM887915/-	Szarowska et al. 2014a
*Dalmatinella fluviatilis* Radoman, 1973	KC344541/-	Falniowski and Szarowska 2013
*Dalmatinella simonae* Beran & Rysiewska, 2021	MT773271/-	Beran et al. 2021
*Ecrobia maritima* (Milaschewitsch, 1916)	KX355835/MG551322	Osikowski et al. 2016/Grego et al. 2017
*Emmericia expansilabris* Bourguignat, 1880	KC810060/-	Szarowska and Falniowski 2013a
*Fissuria boui* Boeters, 1981	AF367654/-	Wilke et al. 2001
*Graecoarganiella parnassiana* Falniowski & Szarowska, 2011	JN202352/-	Falniowski and Szarowska 2011
*Graecoarganiella* sp.	JN202353/MN03140	Falniowski and Szarowska 2011/Hofman et al. 2019
*Graziana alpestris* (Frauenfeld, 1863)	AF367641/-	Wilke et al. 2001
*Grossuana hohenackeri* (Küster, 1853)	KC011749/-	Falniowski et al. 2012
*Hauffenia michleri* (Kuščer, 1932)	KT236156/KY087878	Falniowski and Szarowska 2015 /Rysiewska et al. 2017
*Heleobia maltzani* (Westerlund, 1886)	KM213723/MK609534	Szarowska et al. 2014b/ Falniowski et al. 2019
*Horatia klecakiana* Bourguignat, 1887	KJ159128/-	Szarowska and Falniowski 2014
*Iglica gracilis* (Clessin, 1882)	MH720985/MH721003	Hofman et al. 2018
*Islamia zermanica* (Radoman, 1973)	KU662362/MG551320	Beran et al. 2016/Grego et al. 2017
*Littorina littorea* (Linnaeus, 1758)	KF644330/KP113574	Layton et al. 2014/unpub.
*Lithoglyphus prasinus* (Küster, 1852)	JX073651/-	Falniowski and Szarowska 2012
*Marstoniopsis insubrica* (Küster, 1853)	AF322408/-	Falniowski and Wilke 2001
Moitessieria cf. puteana Coutagne, 1883	AF367635/MH721012	Wilke et al. 2001/Hofman et al. 2018
*Montenegrospeum bogici* (Pešić & Glöer, 2012)	KM875510/MG880218	Falniowski et al. 2014/Grego et al. 2018
*Montenegrospeum sketi* Grego & Glöer, 2018	MG880216/-	Grego et al. 2018
*Paladilhiopsis grobbeni* Kuščer, 1928	MH720991/MH721014	Hofman et al. 2018
*Pontobelgrandiella* sp. Radoman, 1978	KU497024/MG551321	Rysiewska et al. 2016/Grego et al. 2017
*Pseudamnicola pieperi* (Schütt, 1980)	KT710670/-	Szarowska et al. 2016a
*Pseudorientalia* sp.	KJ920477/-	Szarowska et al. 2014c
*Radomaniola curta* (Küster, 1853)	KC011814/-	Falniowski et al. 2012
*Radomaniola curta curta* (Küster, 1853)	KC011781KC011784KC011787KC011788KC011791KC011792KC011810/-	Falniowski et al. 2012
*Radomaniola* sp.	KC011727KC011745KC011747KC011763KC011764KC011766/-	Falniowski et al. 2012
*Sadleriana fluminensis* (Küster, 1853)	KF193067/-	Szarowska and Falniowski 2013b
*Sarajana apfelbecki* (Brancsik, 1888)	MN031432/MN031438	Hofman et al. 2019
Sarajana cf. apfelbecki	MN031431/-	Hofman et al. 2019
*Tanousia zrmanjae* (Brusina, 1866)	KU041812/-	Beran et al. 2015

## Systematic part

### Bivalvia

**Pisidiidae**

#### Pisidium cf. personatum

Taxon classificationAnimaliaSphaeriidaSphaeriidae

A.W. Malm, 1855

0748C838-DB4C-5437-8B5D-009693107737

##### Remarks.

**Figure 4.**
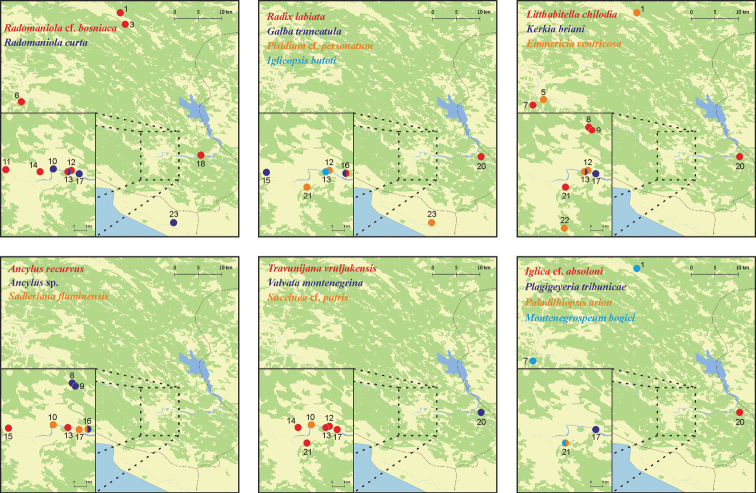
Distribution of the studied taxa. Localities' numbers correspond to Table 1.

Specimens of this common, widely distributed, Holarctic and eurybiotic species were found in many springs. It was also collected from interstitial habitats (with a Bou-Rouch pump) at the localities 12, 21 and 23 (Fig. 4).

### Gastropoda

**Neritopsina: Neritidae**

#### Theodoxus callosus

Taxon classificationAnimaliaCycloneritidaNeritidae

(Deshayes, 1833)

9F419493-D4EA-53FE-AB73-8F308FD0B934

##### Remarks.

This species, described from Greece and reported from Greece and Albania, was found at some larger springs, but never in subterranean waters.

### Caenogastropoda

**Hydrobiidae: Sadlerianinae**

#### Sadleriana fluminensis

Taxon classificationAnimaliaLittorinimorphaHydrobiidae

(Küster, 1852)

F59640E2-8461-531B-8BB8-069D17DA61FB

Fig. 5A

##### GenBank no.

COI: MZ027620–MZ027622

**Figure 5.**
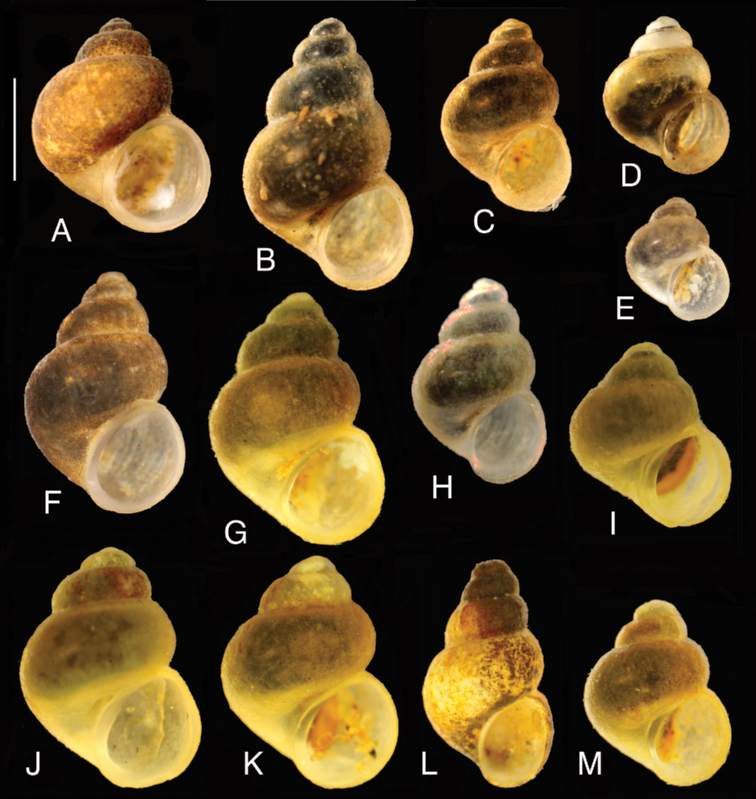
Shells of the studied gastropods: **A***Sadleriana fluminensis*, locality 10 **B–M***Radomaniola***B–H***R. curta* (localities: **B–D** – 10, **E, F** – 13, **G** – 17, **H** – 23) **I–M**R. cf. bosniaca (localities: **I–K** – 1, **L, M** – 12). Scale bar: 1 mm.

##### Remarks.

The most widely distributed species of *Sadleriana*. Found at the localities 10, 16 and 17 (Fig. 4).

#### Radomaniola

Taxon classificationAnimaliaLittorinimorphaHydrobiidae

Szarowska, 2006

E6EE8782-C3C8-570D-BF2B-5B1FAA3486C8

##### Remarks.

**Figure 6.**
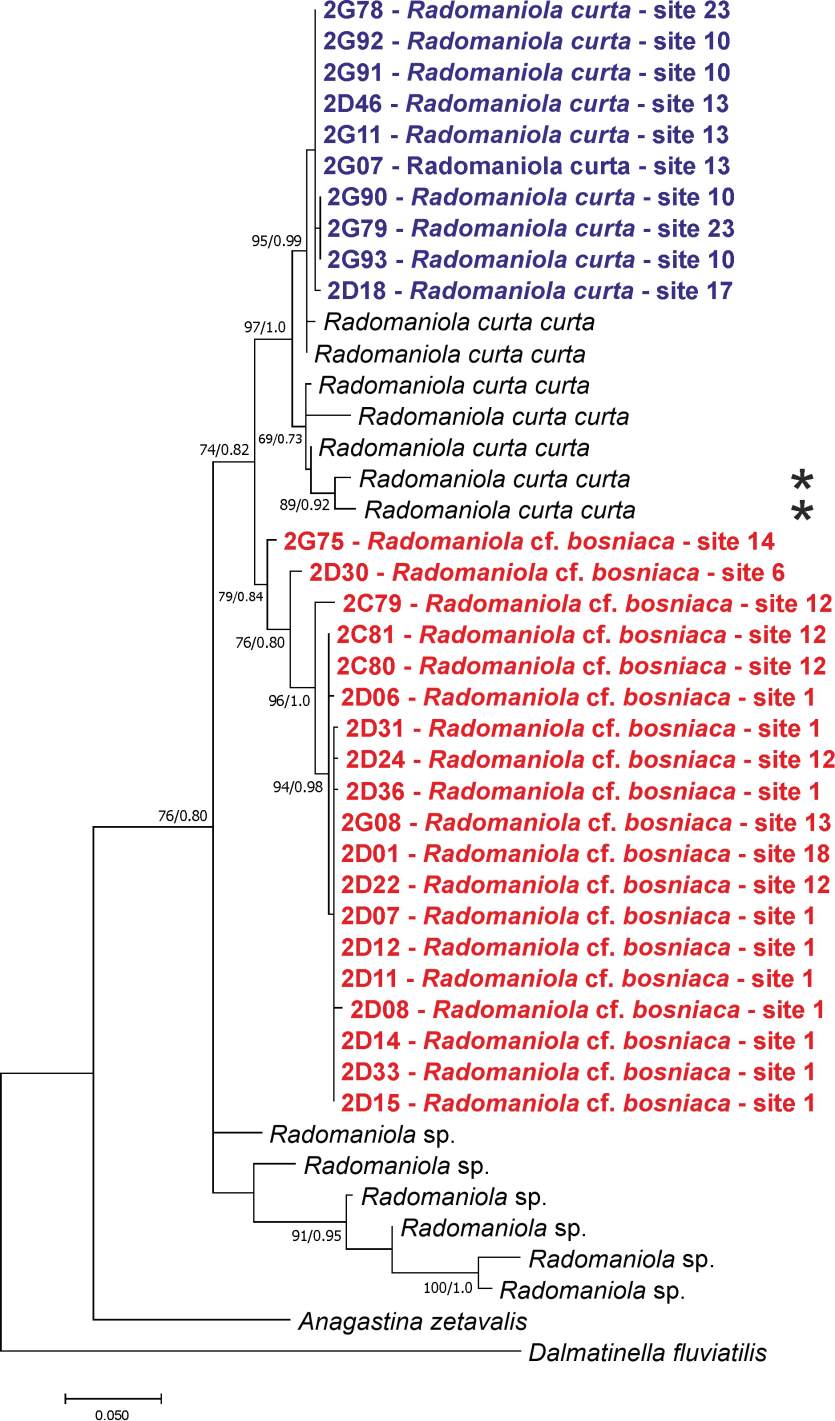
Maximum likelihood (ML) phylogram of the studied *Radomaniola*, based on the partial cytochrome oxidase subunit I (COI) sequences, bootstrap supports given if >60%, together with Bayesian probabilities; topotypes of *R. curta curta* marked with asterisks.

Replacement name for *Orientalina* Radoman, 1978. The genus is widely spread in the former Yugoslavia, but recorded also from Italy. Radoman (1983) distinguished six species of *Radomaniola*, and in one of them – *R. curta* – eight subspecies. It has to be noted that in modern phylogenetics, the only acceptable meaning of a subspecies is a geographic race, which was hardly the case in Radoman’s classification; also, far from being acceptable is that all his species-level taxonomy was based on the shell alone, strikingly variable in this genus (e.g., Falniowski et al. 2012; see also Fig. 5B–M). Molecular and anatomical data (Falniowski et al. 2012) did not confirm the classification of Radoman (1983), but demonstrated high genetic diversity, suggesting a flock of distinct species. The phylogeography as well as molecularly-based species discrimination in *Radomaniola* should be studied with more extensive material, which we are proposing to do. At the moment, considering only *Radomaniola* from the area sampled in this study, one can distinguish two main clades (Fig. 6), representing at least two distinct species. For the one including the sequences of the snails from the spring at Vranjicke Njive, type locality of *Radomaniola curta curta* (sequences KC011781 and KC011784), we used a provisional assignment to this species; for the second clade we provisionally used the name R. cf. bosniaca. In general, the representatives of *Radomaniola* were the most common snails at the studied localities, and were found at the surface, as well as in the pumped interstitial samples and could also be found in caves. *Radomaniola*, pigmented and with eyes, is a stygophile gastropod.

#### Radomaniola curta

Taxon classificationAnimaliaLittorinimorphaHydrobiidae

(Küster, 1852)

637E4472-9410-5863-BC39-262F67D79975

Fig. 5B–H

##### GenBank no.

COI: MW879241–MW879250

##### Remarks.

Found at the localities 10, 13 and 23 (Fig. 4) on the surface and also interstitially and at the locality 17 only on the surface. At the locality 13 in the spring Polički Studenac, in sympatry with R. cf. bosniaca.

#### Radomaniola cf. bosniaca

Taxon classificationAnimaliaLittorinimorphaHydrobiidae

(Radoman, 1973)

B8E1EDBC-15F4-5CF0-B43A-502ACF68DFA1

Fig. 5I–M

##### GenBank no.

COI: MW879222–MW879240

##### Remarks.

Collected at the localities 1, 6, 12, 13, 14 and 18 (Fig. 4) on the surface, but only at the localities 3 and 11 interstitially. At the locality 13 in sympatry with *R. curta*.

#### Kerkia briani

Taxon classificationAnimaliaLittorinimorphaHydrobiidae

Rysiewska & Osikowski, 2020

E6878C2D-C27B-51BC-B5CA-B2D1383E5092

Fig. 7A–C

##### GenBank no.

COI: MT780191–MT780196; H3: MT786730–MT786735; Hofman et al. 2020b

##### Remarks.

Found at the locality 13 (Fig. 4), its type locality, and at locality 17 (about 1 km away), where it is an element of the meiofauna; pumped with a Bou-Rouch pump (Hofman et al. 2020b).

**Figure 7.**
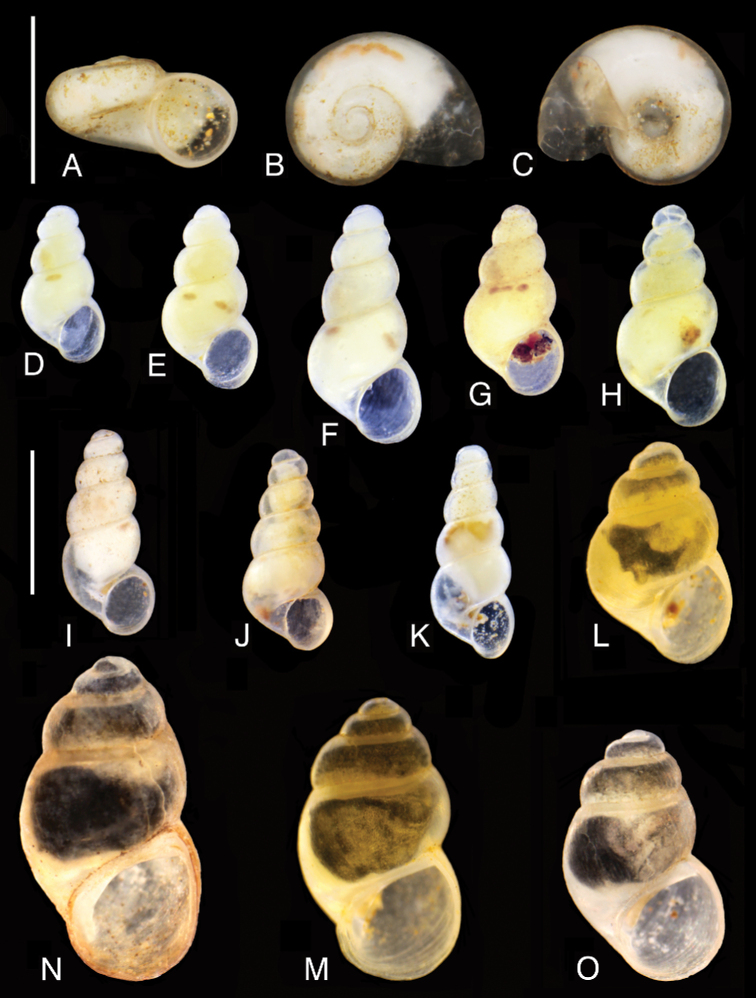
Shells of the studied gastropods: **A–C***Kerkia briani***D–K***Montenegrospeum bogici* (localities: **D–F** – 1, **G, H** – 7, **I** – 13, **J** – 14, **K** – 21) **L–O***Litthabitella chilodia* (localities: **L, M** – 17, **N–O** – 8). Scale bars: 1 mm.

#### Montenegrospeum bogici

Taxon classificationAnimaliaLittorinimorphaHydrobiidae

(Pešić & Glöer, 2012)

0418FAC2-9819-5CA8-B453-DF9FB20235BA

Fig. 7D–K

##### GenBank no.

COI: MZ266648–MZ266650

##### Remarks.

Pešić and Glöer (2012) described a new species of *Bythiospeum* Bourguignat, 1882: *B. bogici* Pešić & Glöer, 2012 from underground waters of Vrelo “Taban”, in central Montenegro. Their description was based on empty shells. Later they (Pešić and Glöer 2013) collected live specimens, and described the lack of eyes and pigment and the penis with a lobe at its medial part. They considered *B. bogici* as belonging to a new genus: *Montenegrospeum* Pešić & Glöer, 2013. Later, Falniowski et al. (2014) demonstrated with molecular data that *Montenegrospeum* belongs to the Hydrobiidae, not Moitessieriidae, despite striking similarity of the shell between this snail and e.g., *Iglica* Wagner, 1927. Numerous live specimens of this species were pumped from interstitial habitats at the localities 1, 7 and 21 (Fig. 4).

#### Litthabitella chilodia

Taxon classificationAnimaliaLittorinimorphaHydrobiidae

(Westerlund, 1886)

27F53EDA-B3C0-5863-B77A-E981B86C24D3

Fig. 7L–O

##### GenBank no.

H3: MZ285059–MZ285063

##### Remarks.

This species was found at the localities 7, 8, 9, 20 and 21 (Fig. 4). It was numerous and was also found in a cave and sometimes interstitially; pumped.

#### Travunijana vruljakensis

Taxon classificationAnimaliaLittorinimorphaHydrobiidae

Grego & Glöer, 2019

A16FD840-75D6-5048-88BE-17C9BED35002

Fig. 9

##### GenBank no.

COI: MW879256–MW879272; H3: MW865737–MW865748

##### Remarks.

Grego and Glöer (2019) described a new monotypic genus *Travunijana* from Vrelo “Goricki Studenac” (Gorica), a spring at the right bank of the Trebišnjica River, this being its type locality. They found it also in two other springs: Vrelo Vruljak 1 (Gorica; our locality 12), and Vrelo Vruljak 3 (Gorica). Their diagnosis of the genus was based on a single “unique” character – the strange morphology of the penis – which was based on artefactual appearance, caused by fixation: a nonglandular outgrowth on the left side, located distally (Grego and Glöer 2019). The penis photographed by them presents a bulbous, drastically contracted distal section, making copulation impossible.

**Figure 8.**
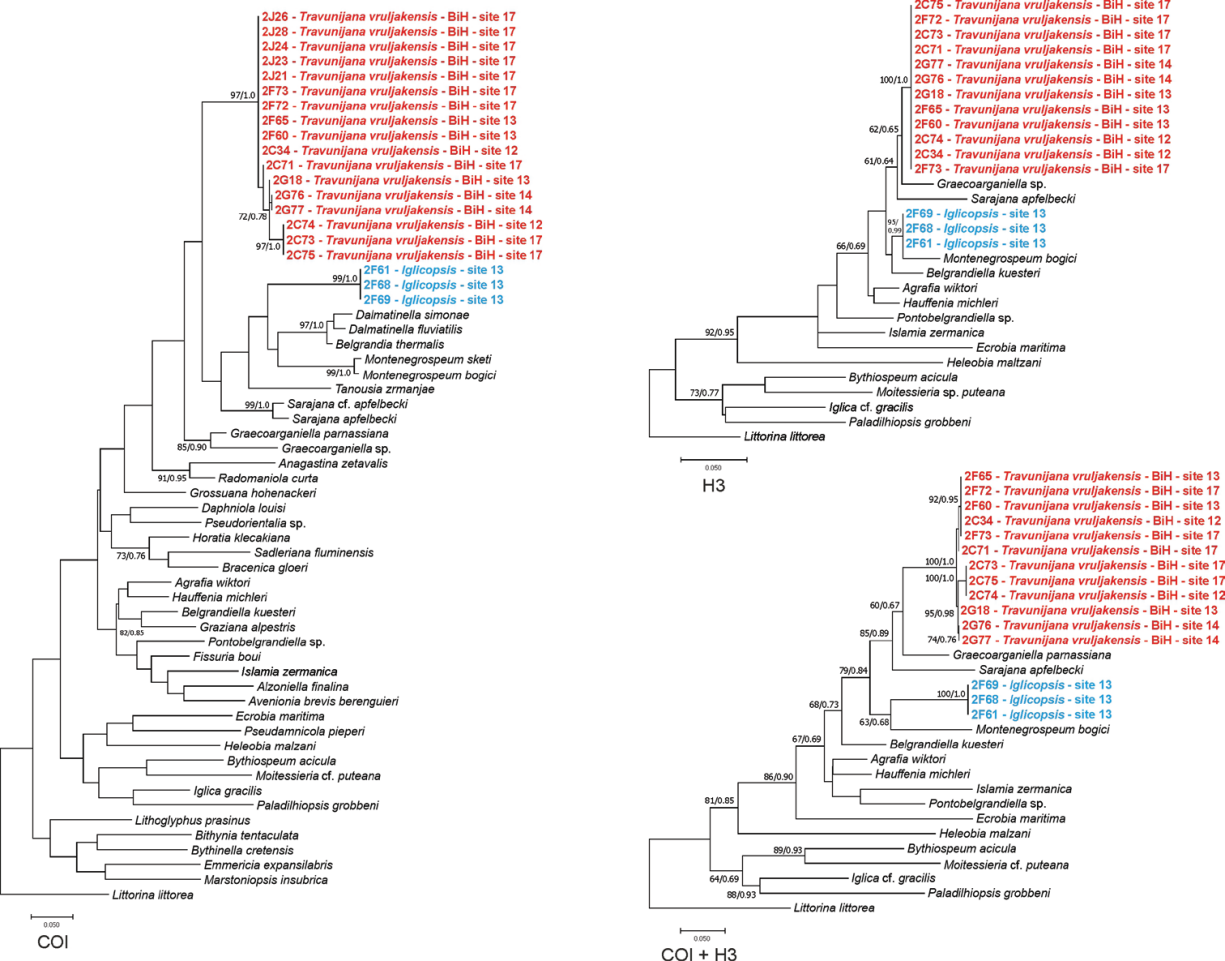
Phylogenetic relationships of *Travunijana* and *Iglicopsis* based on COI, H3 and concatenated loci; bootstrap supports given if over 60%, their values together with Bayesian probabilities.

Our molecular data (Fig. 8) confirmed the distinctiveness of the genus *Travunijana* Grego & Glöer, 2019. The phylograms based on H3, as well as on both concatenated loci placed *Travunijana* as the sister species with *Graecoarganiella* Falniowski & Szarowska, 2011, and *Sarajana* Radoman, 1975 (bootstrap 85%). The shell habitus is different (conic in *Travunijana*, ovate-conic in *Sarajana*), and the penial morphology differs (Hofman et al. 2019): the outgrowth on the left side is simple and filamentous in *Sarajana*, and short and bi-lobed in *Travunijana*. The phylogram based on COI showed a more complicated pattern, but bootstrap supports were too low for any more certain placement in the phylogeny.

#### Redescription of the genus Travunijana

Taxon classificationAnimaliaLittorinimorphaHydrobiidae

Grego & Glöer, 2019

62210712-3F76-514B-9061-B68E88ED3713

##### Diagnosis.

Shell conic with moderately convex whorls, big sphaerical bursa copulatrix and two nearly vestigial receptacula seminis, penis long and slender, distally forming a slightly bent filament, at the base of the filament an outgrowth on the left side of the penis, formed of two finger-like lobes.

##### Description.

**Figure 9.**
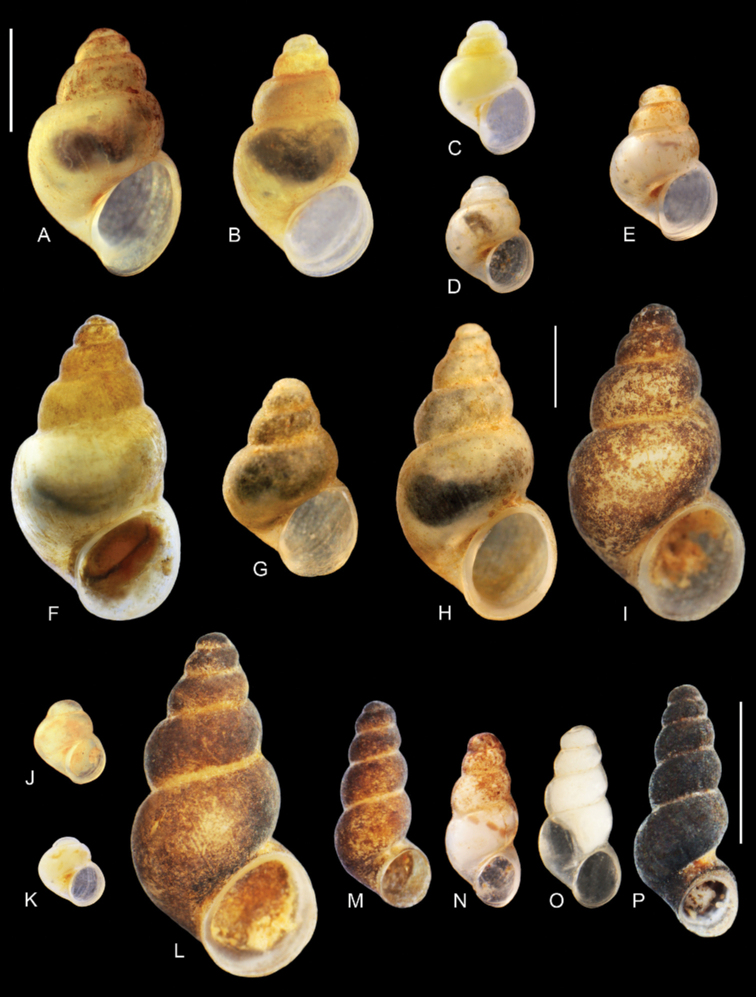
Shells: **A–L***Travunijana vruljakensis***M–P***Iglicopsis butoti***M** holotype **N** 2F61 **O** 2F68 **P** 2F69 (extraction numbers, see Table 3). Scale bars: 1 mm.

**Figure 10.**
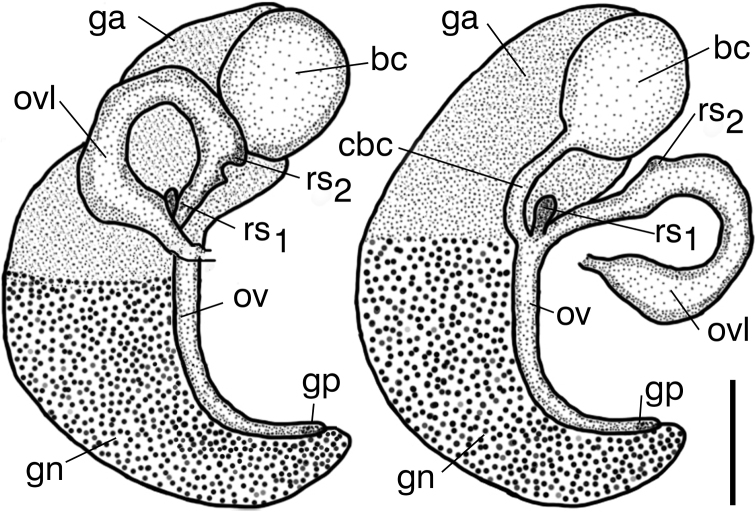
Female reproductive organs of *Travunijana vruljakensis* (bc – bursa copulatrix, cbc – duct of bursa, ga – albuminoid gland, gn – nidamental gland, gp – gonoporus, ov – oviduct, ovl – loop of (renal) oviduct, rs_1_ – distal seminal receptacle, rs_2_ – proximal seminal receptacle). Scale bar: 0.25 mm.

**Figure 11.**
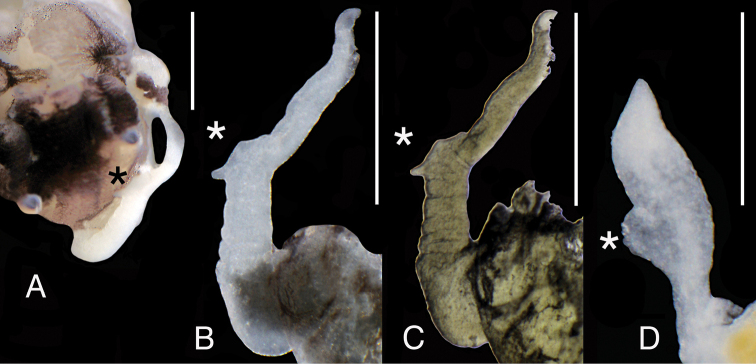
Penis of *Travunijana vruljakensis*. Scale bars: 0.5 mm.

The shell (Fig. 9) as described by Grego and Glöer (2019). The female reproductive organs (Fig. 10) with bulbous loop of (renal) oviduct, big and spherical bursa copulatrix and two nearly vestigial receptacula seminis: proximal (rs_2_ of Radoman 1973) and distal (rs_1_ of Radoman 1973) one. The penis (Fig. 11) long and slender, slightly bent at its medial section, at the base of the long filamentous distal section and an outgrowth on the left side, consisted of two finger-like lobes.

*Travunijana vruljakensis* was common at the studied territory, found at the localities 12, 13, 14, 17 and 21. At 12, 13 and 17 (Fig. 4) interstitially pumped.

#### Iglicopsis

Taxon classificationAnimaliaLittorinimorphaHydrobiidae

Falniowski & Hofman gen. nov.

51081D01-6085-5D24-A821-4582CEA617E6

http://zoobank.org/77758877-EEF4-448E-B727-D5632F9E5F51

##### Type species.

*Iglicopsis butoti* by original designation

##### Diagnosis.

Shell ovate-conic with broad and flat apex, transparent, operculum smooth, no pigment, eyes absent, ctenidium present, penis long, tapering, with bi-lobed outgrowth on the left side and flat outgrowth at the right side, unpigmented renal oviduct, bursa copulatrix and two small receptacula seminis.

##### Remarks.

*Iglicopsis* shell resembles that of *Montenegrospeum*, but is more oval, with broader spire and broader flat apex, sometimes showing scalarity at the body whorl; the penis with the left-side outgrowth located more proximally and bi-lobed and additional flat outgrowth on the right side; the molecular divergence (p = 0.186 for mitochondrial COI and p = 0.114 for nuclear H3) at the genus-level.

#### Iglicopsis butoti

Taxon classificationAnimaliaLittorinimorphaHydrobiidae

Falniowski & Hofman sp. nov.

E38D4FA4-D7D4-5A4F-9432-FD2EA6679075

http://zoobank.org/C1A9D0B0-4B10-4977-B69B-7C4C42BB19D3

Fig. 9M–P

##### GenBank no.

COI: MW879273–MW879275; H3: MW865749–MW865751

##### Type materials.

***Holotype.*** Ethanol-fixed specimen (Fig. 9M), Vrelo „Polički Studenac” (Crkvina); a cave spring in the left bank of and adjacent to the Trebišnjica River (N 42.71288, E 18.36514) (our locality 13, Fig. 4) close to Trebinje (Bosnia and Herzegovina), interstitially, 50 cm below the gravel floor of the spring; in the collection of the Department of Malacology of Jagiellonian University, voucher number ZMUJ-M.2651.

***Paratypes.*** Three paratypes destroyed to extract DNA, one specimen ethanol-fixed, in the collection of the Department of Malacology of Jagiellonian University, ZMUJ-M.2652.

##### Diagnosis.

Shell minute, ovate-conic, distinguishable from *Montenegrospeum* by a more oval habitus, broader spire and broader flat apex, sometimes showing scalarity at the body whorl; the penis with the left-side outgrowth located more proximally and bi-lobed, and additional flat outgrowth on the right side.

##### Description.

*Shell* (Fig. 9M–P) up to 1.49 mm high and 0.55 mm broad, ovate-conic, whitish, translucent, thin-walled, and consisting of about five whorls, growing regularly and separated by moderately deep suture. Spire high and broad, apex broad and flat, body whorl less than 0.5 of the shell height, Aperture small, prosocline, oval in shape, peristome complete and thin, somewhat swollen, in contact with the wall of the body whorl, in some specimens showing scalarity close to the aperture, umbilicus slit-like. Shell surface smooth, with growth lines hardly visible.

*Measurements* of holotype and sequenced and illustrated shells: Table 3. Shell variability slight; scalarity and much bigger dimensions of one specimen (Fig. 9P) most probably caused by the larval Trematoda (parasite gigantism).

**Table 3.** Shell measurements (in mm) of holotype and sequenced and illustrated specimens of *Iglicopsis butoti* sp. nov. For explanation of the symbols *a*-β, see Fig. 13B.

	**Holotype**	**2F61**	**2F68**	**2F69**
*a*	1.49	1.29	1.35	1.87
*b*	0.55	0.54	0.54	0.70
*c*	0.43	0.39	0.43	0.44
*d*	0.80	0.62	0.67	0.93
*e*	0.37	0.34	0.35	0.44
α	90	89	90	90
β	20	18	20	18

*Soft parts morphology and anatomy*. Body white, pigmentless, with no eyes. Ctenidium with nine short lamellae, osphradium elongated. Tectum forming a characteristic broad loop (Fig. 9N). Female reproductive organs with unpigmented renal oviduct, bursa copulatrix and two small receptacula seminis; details unknown.

**Figure 12.**
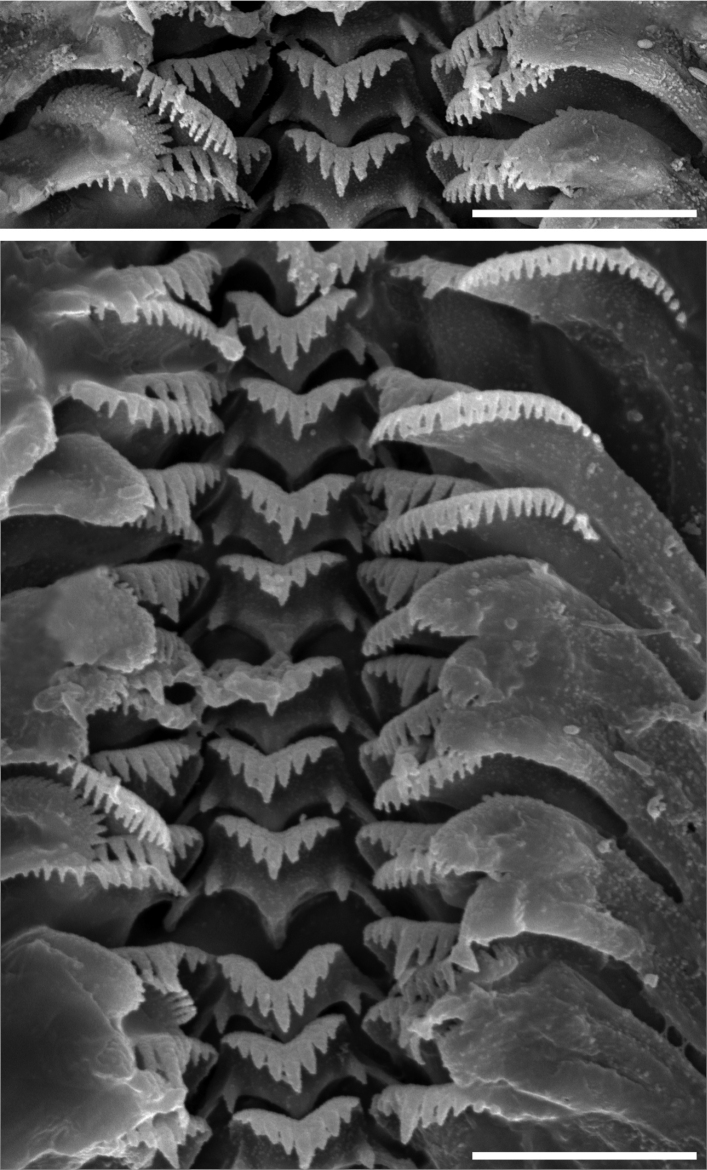
Radula of *Iglicopsis butoti*, scale bars: 10 µm.

The radula (Fig. 12) with the central tooth cusp formula:

$\frac{\left(4)3-1-3(4\right)}{1-1}$ or $\frac{\left(5)4-1-4(5\right)}{1-1}$

Rather long and slender cusps grow regularly to central one. Lateral cusp with 5 – 1 – 5(6) long and massive cusps. Inner marginal tooth with ca 23 slender cusps of nearly invariable length along the tooth edge, outer marginal tooth with 26 broadly triangular cusps.

**Figure 13.**
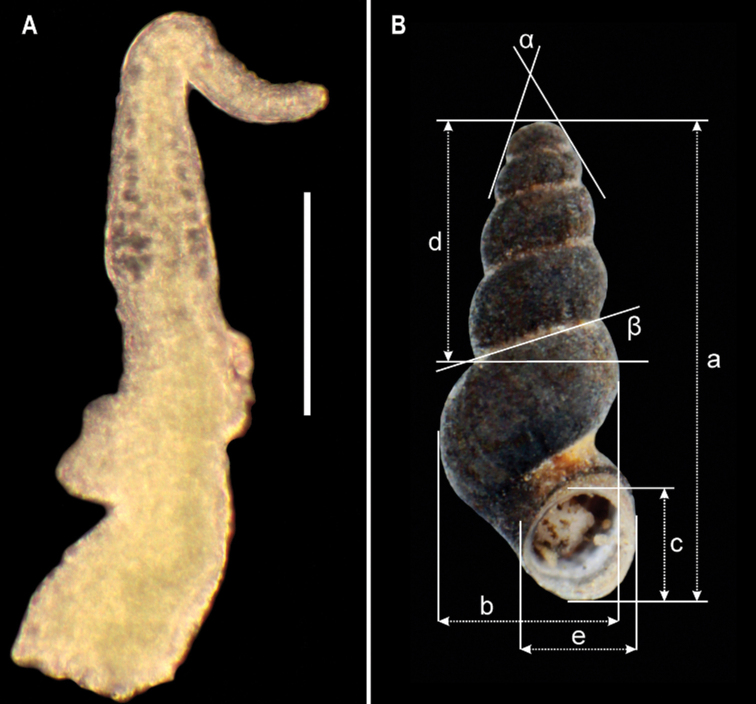
**A** Penis of *Iglicopsis butoti*, scale bar: 0.1 mm **B** shell measurements: *a* – shell height, *b* – body whorl breadth, *c* – aperture height, *d* – spire height, *e* – aperture breadth, α – apex angle, β – angle between body whorl suture and horizontal surface.

Penis (Fig. 13A) long, tapering, below the half of its length, proximally, bi-lobed outgrowth on the left side and flat outgrowth at the right side, at the distal part and the vas deferens well visible inside, running in zigzags.

##### Derivatio nominis.

The genus name refers to the similarity of the shell to the moitessieriid genus *Iglica* Wagner, 1927. The specific epithet *butoti* refers to the memory of Dr Louis J. M. Butot, a Dutch malacologist devoted mostly to the Greek malacofauna, good friend and the mentor of AF.

##### Distribution and habitat.

Known from the type locality only.

##### Molecular relationships.

despite its shell morphology, *Iglicopsis* clearly belongs to the Hydrobiidae Stimpson, 1865, Sadlerianinae Szarowska, 2006, and not to the Moitessieriidae Bourguignat, 1863 (Fig. 8). Its sister species is *Montenegrospeum bogici* in the H3 tree (Fig. 8, bootstrap 95%), and on the tree based on both concatenated loci (but with bootstrap 63% only); in the COI tree the bootstrap does not support its phylogenetic position.

### Emmericiidae

#### Emmericia ventricosa

Taxon classificationAnimaliaLittorinimorphaEmmericiidae

Brusina, 1870

3466D4BE-A1E3-542C-BE62-4A8F210A9AA4

Fig. 14A–C

##### GenBank no.

COI: MZ027623–MZ027627

##### Remarks.

The species was found at the localities 1, 5, 12, 13, 22 (estavelle) (Fig. 4), at the surface. Molecular data rather confirms its distinctiveness (p = 0.038) from *E. expansilabris* (Bourguignat, 1870), described from Vrelo “Ombla” on the Dalmatian coast in nearby Croatia.

**Figure 14.**
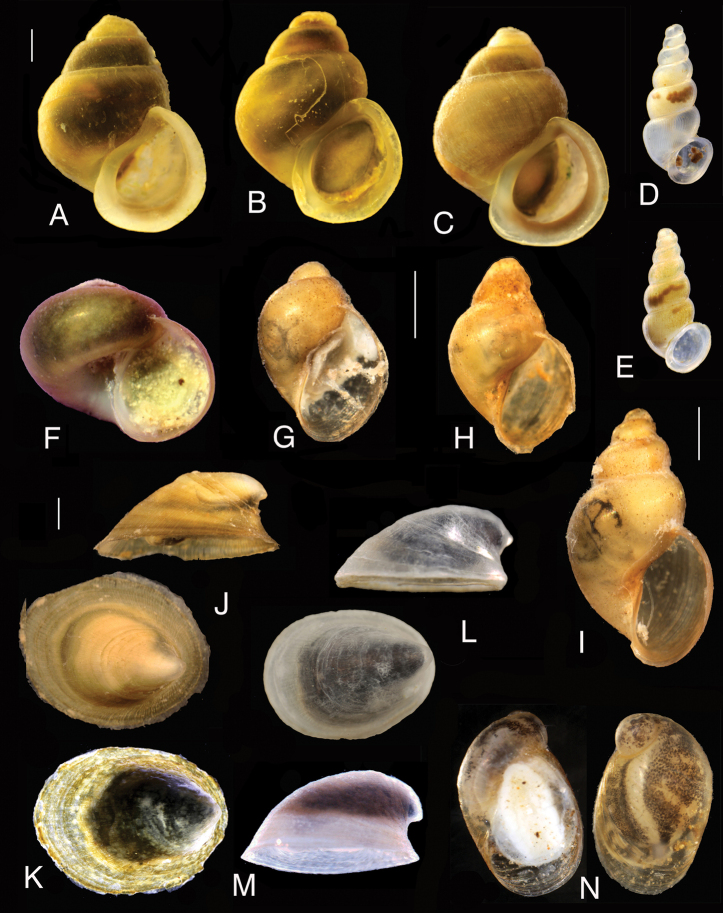
Shells of the studied gastropods: **A–C***Emmericia ventricosa* (localities: **A** – 1, **B** – 5, **C** – 12) **D, E***Paladilhiopsis arion* (locality 21) **F***Valvata montenegrina* (locality 20) **G***Radix labiata* (locality 16) **H, I***Galba truncatula* (localities: **H** – 15, **I** – 16) **J, K***Ancylus recurvus* (localities: **J** – 15, **K** – 13) **L, M***Ancylus* sp. C4 (localities: **L** – 9, **M** – 16) **N**Succinea cf. putris (locality 10). Scale bars: 1 mm.

### Moitessieriidae

#### Iglica cf. absoloni

Taxon classificationAnimaliaLittorinimorphaHydrobiidae

(A.J. Wagner, 1914)

3050E832-10B3-59C6-9A86-F0374E440915

##### Remark.

Empty shell was found interstitially at the locality 20 (Fig. 4).

#### Plagigeyeria tribunicae

Taxon classificationAnimaliaLittorinimorphaHydrobiidae

Schütt, 1963

A4FD9C3A-24DA-552B-BFBA-23373D9CD8E9

##### Remark.

Empty and incomplete shell was found interstitially at the locality 17 (Fig. 4).

#### Paladilhiopsis arion

Taxon classificationAnimaliaLittorinimorphaHydrobiidae

Rysiewska & Osikowski, 2021

3A269622-26B6-5707-AD86-F16AAB058BD6

Fig. 14D, E

##### GenBank no.

COI: MW741739–MW741740; H3: MW776424–MW776425

##### Remarks.

Live specimens were pumped from an interstitial habitat at the locality 21 (Fig. 4). They were recently described as new to science (Hofman et al. 2021). Morphologically and molecularly, they were distinct from the moitessieriid species discussed in Hofman et al. (2018). Rysiewska et al. (2021) demonstrated that at least some of the species assigned to the genus *Plagigeyeria* Tomlin, 1930 belong to the genus *Paladilhiopsis* Pavlović, 1913. Our specimens from Gučina in Trebinje molecularly formed the sister clade with *Plagigeyeria montenegrina* Bole, 1961 from Obodska Pečina in Montenegro. Also, the outline and orientation of the long axis of the aperture was characteristic of *Plagigeyeria*. The similarly shaped shell and geographic range may suggest assignment to *P. nitida* Schütt, 1963, but the number of whorls of our specimens is much higher than presented by Schütt (1972).

### Heterobranchia

**Heterostropha: Valvatidae**

#### Valvata montenegrina

Taxon classificationAnimaliaHeterostrophaValvatidae

Glöer & Pešić, 2008

DD010EB0-FCB3-5591-BCF1-8F7528C0A036

Fig. 14F

##### GenBank no.

COI: MZ027632–MZ027633

##### Remark.

Some specimens found at the locality 20 (Fig. 4); in the surface waters.

### Pulmonata

**Lymnaeidae**

#### Radix labiata

Taxon classificationAnimaliaHygrophilaLymnaeidae

(Rossmässler, 1835)

A1A38D69-4A75-54EA-AAB4-86792A22BCA7

Fig. 14G

##### GenBank no.

COI: MZ027630

##### Remarks.

This common Central-European and Mediterranean species was found at the localities 16 and 20 (Fig. 4). Inhabits slowly running or stagnant small water bodies (e.g., Glöer 2019), preferably close to ground waters, but not found in subterranean habitats.

#### Galba truncatula

Taxon classificationAnimaliaHygrophilaLymnaeidae

(O. F. Müller, 1774)

5E74A8F1-CC6B-5F2B-A9E6-F6165138EC7E

Fig. 14H, I

##### GenBank no.

COI: MZ027628–MZ027629

##### Remarks.

Common Palaearctic gastropod, inhabiting nearly all of Europe. This amphibious and calcifilous (e.g., Glöer 2019) species inhabits small water bodies, rich in vegetation, such as at our locality 16 – a small lake in a collapsed cave, rather than subterranean habitats, but at the locality 15 it was found in an estavelle, a kind of vast subterranean tunnel transporting water either down, as outlet of surface waters, or up, forming temporary active springs. Shells of our specimens (Fig. 14H, I) were somewhat untypical, with low and broad spire, but the variation of the shell in the Lymnaeidae has been long known (e.g., Roszkowski 1914; Falniowski 1980, 1981), as being wider than in any other gastropod group.

### Ancylidae

#### Ancylus recurvus

Taxon classificationAnimaliaHygrophila Ancylidae

Martens, 1783

7D19D83F-EF8F-508E-AAB9-78E06135DC24

Fig. 14J, K

##### GenBank no.

COI: MW879251–MW879253

##### Remarks.

**Figure 15.**
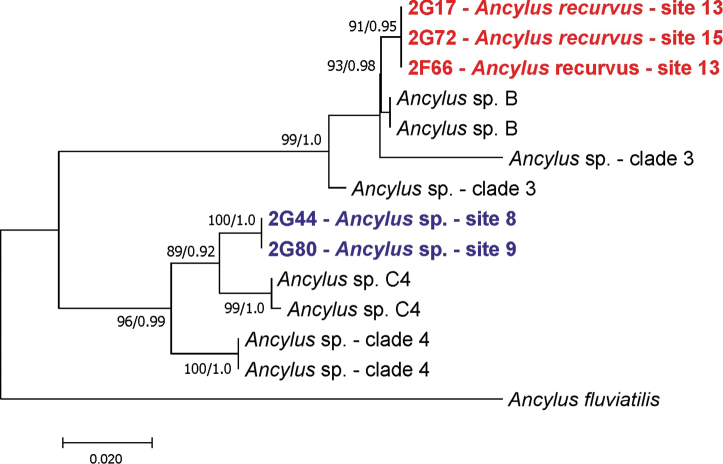
Molecular relationships of the studied *Ancylus* based on COI; our sequences in red and orange, the other from GenBank; bootstrap supports given if over 60%, their values together with Bayesian probabilities.

*Ancylus* is known as a stygophile gastropod (e.g., Culver and Pipan 2009; Macher et al. 2016; personal observations); also inhabiting caves. *Ancylus recurvus* at the locality 13 was also found interstitially, pumped, and at the locality 15 (Fig. 4) it inhabited an estavelle. Our *A. recurvus* molecularly belonged to the clade “*Ancylus* sp. B” of Albrecht et al. (2006), Clade 3 of Pfenninger et al. (2003) (Fig. 15). It is molecularly different from *A. fluviatilis* by 9%.

#### Ancylus

Taxon classificationAnimaliaHygrophilaAncylidae

sp.

17B00DC2-33D2-527C-9A43-31701A60A255

Fig. 14L, M

##### GenBank no.

COI: MW879254–MW879255

##### Remarks.

Considering the shell morphology, it should be determined as *A. fluviatilis* O. F. Müller, 1774, a species reported from this region. However, Pfenninger et al. (2003) demonstrated that *A. fluviatilis* inhabits a wide range throughout Europe, but in the southern regions there are a few cryptic, molecularly defined species of *Ancylus*. Our *Ancylus* sp. molecularly belonged to the Clade 4 of Pfenninger et al. (2003) and “*Ancylus* sp. C4” of Albrecht et al. (2006) (Fig. 15). It was found as a crenobiont in the cave springs at the localities 8, 9 and 16 (Fig. 4). Molecular divergence between this *Ancylus* sp. and *Ancylus recurvus* is 7%, and similar value (7.5%) is observed between this *Ancylus* sp. and *A. fluviatilis*.

### Stylommatophora: Succineidae

#### Succinea cf. putris

Taxon classificationAnimaliaStylommatophoraSuccineidae

(Linnaeus, 1758)

C173DB00-7626-57E4-9346-E889062512D0

Fig. 14N

##### GenBank no.

COI: MZ027631

##### Remarks.

Our specimen differed by 12 substitutions (97.55% of identity) from *Succinea* sp. GenBank number KF412772 from “Egypt: Fayoum Governorate”. For the closest European *Succinea*, *S. putris* the identity was only 86.73%. In fact, this value is close to the threshold one to distinguish species in the Pulmonata, thus our specimen may represent some still unsequenced species of *Succinea*. This amphibious snail was found at locality 10 (Fig. 4).

## Supplementary Material

XML Treatment for Pisidium cf. personatum

XML Treatment for Theodoxus callosus

XML Treatment for Sadleriana fluminensis

XML Treatment for Radomaniola

XML Treatment for Radomaniola curta

XML Treatment for Radomaniola cf. bosniaca

XML Treatment for Kerkia briani

XML Treatment for Montenegrospeum bogici

XML Treatment for Litthabitella chilodia

XML Treatment for Travunijana vruljakensis

XML Treatment for Travunijana

XML Treatment for Iglicopsis

XML Treatment for Iglicopsis butoti

XML Treatment for Emmericia ventricosa

XML Treatment for Iglica cf. absoloni

XML Treatment for Plagigeyeria tribunicae

XML Treatment for Paladilhiopsis arion

XML Treatment for Valvata montenegrina

XML Treatment for Radix labiata

XML Treatment for Galba truncatula

XML Treatment for Ancylus recurvus

XML Treatment for Ancylus

XML Treatment for Succinea cf. putris
